# Diagnosis and treatment of giant colonic fecalith in a child: a case report

**DOI:** 10.3389/fped.2025.1598807

**Published:** 2025-05-13

**Authors:** Wei Su, Jing Chen, Yuxia Zhao, Pingping Xu, Jingwen Yuan, Chunqi Luo, Jie Liu, Baoxiang Wang

**Affiliations:** Wuhan Children’s Hospital, Tongji Medical College, Huazhong University of Science and Technology, Wuhan, China

**Keywords:** fecalith, intestinal obstruction, phytobezoar, colonic stone, manual extraction

## Abstract

Fecaliths are concretions composed of undigested or partially digested substances formed within the gastrointestinal lumen, potentially causing obstruction or partial obstruction. The most common type of fecalith is phytobezoar, composed of plant fibers. Due to their high cellulose, hemicellulose, and lignin content, phytobezoars remain undigested within the stomach and intestines, causing blockage in the narrowest portions of the gastrointestinal tract. Obstructions caused by fecaliths typically occur in the stomach and small intestine, whereas colonic obstructions are rare, particularly in pediatric cases. In this study, we report for the first time a case of colonic obstruction caused by a giant fecalith measuring 6 cm × 6 cm, which was successfully managed by colonoscopy-assisted manual extraction under general anesthesia.

## 1 Introduction

Fecaliths are concretions formed by aggregating debris or gastric contents within the gastrointestinal tract. Based on their composition, they are classified into phytobezoars, composed of plant fibers; trichobezoars, composed of hair; lactobezoars formed by infants continuously consuming concentrated milk formula; and pharmacobezoars formed from extended-release medications, among others (1). Among these types, phytobezoars are the most common, with high-fiber diets being a primary contributing factor. Complications associated with fecaliths include mechanical irritation and gastrointestinal obstruction, with severe cases potentially progressing to gastric perforation and resultant peritonitis. Fecalith-induced obstructions account for approximately 0.4%–4% of all gastrointestinal obstructions, predominantly occurring in the stomach or small intestine. Colonic obstruction due to fecaliths is considered extremely rare (2). In this study, we diagnosed a pediatric colonic obstruction caused by a giant phytobezoar, which was successfully treated via colonoscopy-assisted manual extraction. This report provides an analysis of the diagnostic and therapeutic process involved in this case.

## 2 Case report

### 2.1 Ethical statement

This study received approval from the Ethics Committee of Wuhan Children'sHospital (No. 2021R064-E04), and informed consent was obtained from thepatient's families of the patient for access to clinical data.

### 2.2 Case report

The patient, a 7-year-old male, was admitted with complaints of recurrent abdominal pain and distension for half a month. He had a prolonged history of constipation and strained defecation. Approximately two weeks earlier, following ingestion of a large amount of unshelled broad beans, the patient experienced recurrent episodes of abdominal pain accompanied by pronounced distension. He had no spontaneous bowel movements, and medication-assisted bowel evacuation had been ineffective. There was no prior history of underlying medical conditions.

Physical examination revealed abdominal distension, hyperactive bowel sounds, and a palpable, hard, egg-sized mass above the pubic symphysis with limited mobility. Digital rectal examination identified a hard mass with smooth edges palpable at the fingertip within the distal rectum.

The peripheral blood routine test showed white blood cells at 9.42 (10^9^/L), hemoglobin 139 (g/L), platelet count 311 (10^9^/L), and neutrophil percentage at 65.4 (%). Blood gas analysis indicated HCO3− at 21 mmol/L, pH at 7.48, and base excess (BE) at −6 mmol/L. Serum biochemical analysis, inflammatory markers, and other blood tests revealed no abnormalities.

**Figure 1:**
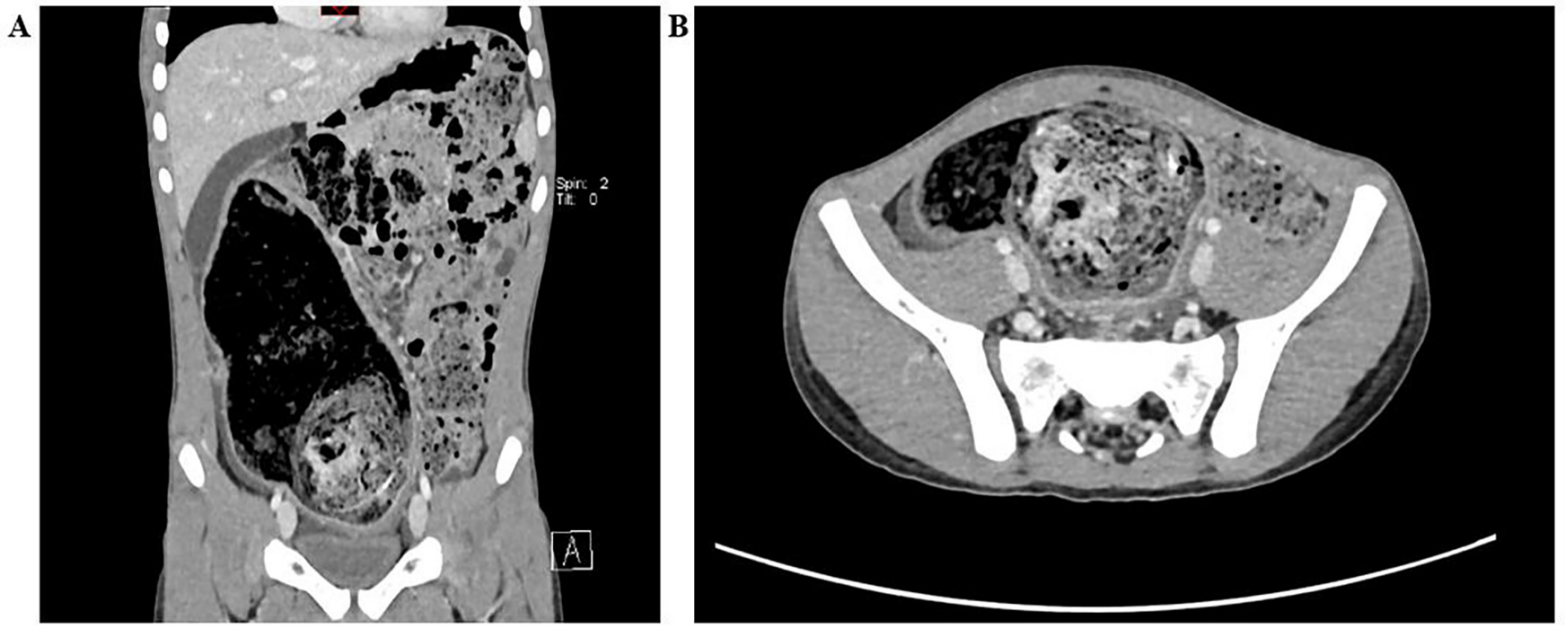
**(A)** Coronary view showing distended large bowel loops up to the distal descending colon where a well-defined ovoid intraluminal mass with a mottled gas pattern is seen. **(B)** ransverse view showing a large mass in a circular cavity of the sigmoid colon with a dilated loop of the large intestine.

**Figure 2:**
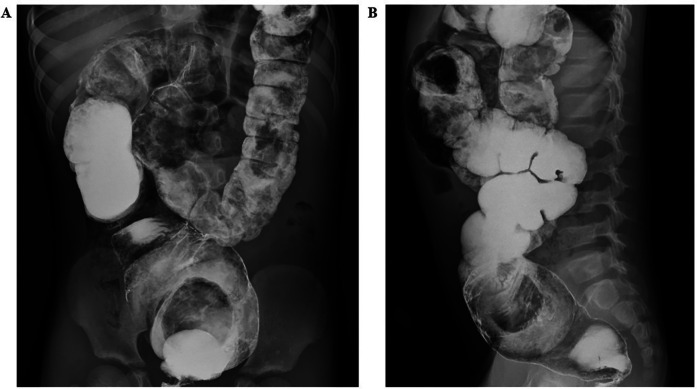
A barium enema examination (**A** and **B** are respectively the anteroposterior and lateral X-ray films of the abdomen) revealed a large filling defect about 6 cm in diameter at the junction of the sigmoid colon and rectum, almost complete obstruction of the colon, resulting in fecal retention.

Abdominal radiographs suggested possible intestinal obstruction. Subsequent abdominal computed tomography (CT) demonstrated fecal retention within the colon and rectum, with an intraluminal mass approximately 6 cm in diameter located at the distal sigmoid colon, consistent with a fecalith (Figure 1). Treatment with colonic dialysis and pharmacologically assisted defecation facilitated the evacuation of considerable muddy stools, alleviating the patient's abdominal pain symptoms somewhat. Follow-up barium enema imaging revealed dilation of the sigmoid colon and rectum, with a large filling defect at the distal sigmoid colon (Figure 2). The fecalith showed no significant change in size or position compared with previous imaging studies.

**Figure 3:**
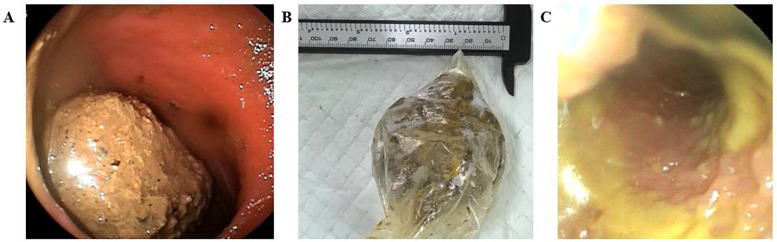
**(A)** Impacted bezoar seen with complete obstruction is seenon colonoscopy. **(B)** A 6 cm × 6 cm round-shaped bezoar was removed from the colon. **(C)** The intestinal cavity of the colon after removal of the fecalith.

Due to persistent abdominal pain symptoms, a colonoscopic examination was performed. Colonoscopy revealed a substantial amount of adherent stool within the lumen, dilation of the sigmoid colon and rectal lumen, and a spherical fecalith (6 cm × 6 cm) at the junction of the sigmoid colon and rectum, occupying nearly the entire lumen (Figure 3A). Attempts were made to extract the fecalith using various colonoscopic instruments (e.g., snare, biopsy forceps, oval forceps). However, extraction proved challenging due to the fecalith's hard consistency, smooth surface, difficulty in stabilization, and resistance to fragmentation.

Subsequently, the patient received three days of paraffin oil enemas and saline enemas aimed at lubricating the intestinal wall and softening the fecalith. Under general anesthesia, another colonoscopy was performed. Initial anal dilation was conducted, and a manual-assisted procedure followed: one hand applied downward abdominal pressure to immobilize and secure the fecalith, while the other hand, cooperating with digital rectal examination, gradually fragmented the hard fecalith into smaller portions, eventually extracting it piece by piece (total weight approximately 94 g, Figure 3B). A repeat colonoscopy reached up to the transverse colon, visualizing a dilated lumen filled with loose fecal matter (Figure 3C). Approximately 150 ml of paraffin oil was instilled via rectal enema, resulting in the evacuation of foul-smelling loose stools. Finally, a colonoscopy confirmed intestinal wall integrity, and the procedure was completed.

Postoperatively, the patient experienced relief from abdominal distension and pain. A follow-up abdominal radiograph indicated significant improvement in colon dilation. Follow-up after discharge revealed the patient maintained regular bowel movements, passing soft-formed stools 1–2 times per day.

## 3 Discussion

Clinically, fecaliths generally present as lower abdominal masses, often leading to misdiagnosis as colonic tumors. Primary colonic fecaliths are exceedingly rare, as fecaliths commonly accompany conditions such as intestinal diverticula, tumors, or postoperative complications causing reduced intestinal motility (3). The predilection parts of fecaliths are the stomach and small intestine, and colonic fecaliths are extremely rare. Yoon et al. reported a case of phytobezoar formation at the rectosigmoid junction following ingestion of plant material in a patient (4). Law et al. reported a case of a distal sigmoid obstruction caused by a phytobezoar in a 60-year-old man with no obvious precipitating causes (5). Currently, reports on fecaliths in pediatric populations are scarce and limited. One study indicated that congenital gastrointestinal malformations, pica, and intellectual developmental delay are primary etiological factors in pediatric fecalith formation, with the stomach as the most common site and phytobezoars as the predominant type (6). A study of gastrointestinal fecalith cases in children from 1980 to 2018, showed the largest number of cases came from the Eastern Mediterranean and the Middle East, possibly due to a diet rich in fruits and vegetables with indigestible seed phytobezoars blocking the intestinal tract. The majority of affected children are male (65%) and range in age from 2 to 16 years, the median age was 10 years (7). In this report, the pediatric patient presented with a significantly large fecalith located at the distal sigmoid colon. Abdominal CT, colonoscopy, and gastrointestinal contrast imaging did not indicate any congenital gastrointestinal malformations. Thus, a diagnosis of primary colonic fecalith was considered, representing the first reported case of a giant colonic fecalith in a child. Chronic constipation and impaired bowel motility, leading to weakened gastrointestinal function, were considered contributing factors. The patient's symptoms began following ingestion of a large amount of inadequately chewed, unshelled broad beans, resulting in the formation of a phytobezoar, which subsequently became impacted at the physiologically narrowed sigmoid-rectal junction, causing intestinal obstruction.

Abdominal pain is the most common clinical symptom of colonic fecaliths, and other symptoms are mostly abdominal distension, vomiting, constipation, palpable masses, loss of appetite, and weight loss (8). Clinical symptoms of colonic fecaliths in children are often atypical, and diagnosis can be challenging due to limited cooperation during physical examination and unclear self-reported symptoms by pediatric patients, making missed or incorrect diagnoses common. Auxiliary examinations are thus crucial for accurate diagnosis. Conventional abdominal radiographs often fail to detect fecaliths clearly due to their radiolucent characteristics. Abdominal CT exhibits sensitivity and specificity of 90% and 57%, respectively, for diagnosing fecalith-induced intestinal obstruction (9). Typical CT findings include round or oval-shaped intraluminal masses of heterogeneous density with sieve-like hypodense areas or gas bubbles (10). Enhanced CT scans additionally allow assessment of bowel vascular perfusion and identification of potential ischemic intestinal changes. In the present case, enhanced abdominal CT clearly delineated the size and location of the fecalith and confirmed adequate blood supply to the surrounding intestinal walls. Thus, the combination of medical history, clinical presentation, and abdominal CT imaging enables early diagnosis of fecalith-induced intestinal obstruction in children, avoiding missed diagnoses or misdiagnoses.

In general, fecaliths pose minimal risks and are typically expelled naturally via bowel movements. However, fecaliths can occasionally become extremely hard, causing intestinal obstruction that disrupts gastrointestinal function, creating a cycle of impaired digestion and absorption. Severe obstruction may lead to intestinal necrosis or even peritonitis, causing critical complications if not promptly identified and treated. Conservative treatment and surgical intervention represent two primary therapeutic approaches for fecalith-induced obstruction. Management varies depending on the fecalith's location, size, and associated complications. Conservative treatments, such as enemas and oral laxatives, are the first-line therapies for fecaliths situated in the distal colon. If conservative treatments fail, colonoscopic removal may be attempted. Surgical intervention is warranted in the presence of complications such as strangulated bowel obstruction, gastrointestinal bleeding, or peritonitis.

The patient in our case had no underlying disease, and the obstruction occurred at the sigmoid-rectal junction—a physiologically narrow region. Given the patient's young age, large fecalith size, and absence of complications such as strangulated bowel obstruction or peritonitis, conservative treatment was initially chosen. After multiple unsuccessful conservative treatment attempts, colonoscopic removal was undertaken. With prior softening of the fecalith using repeated paraffin oil and saline enemas, a colonoscopic-assisted manual extraction was successfully performed under general anesthesia, avoiding surgical trauma.

## 4 Conclusion

In summary, this case provides valuable clinical experience regarding the diagnosis and treatment of giant colonic fecalith in a pediatric patient. Colonoscopic-assisted, minimally invasive manual extraction under general anesthesia is recommended as a safe and effective primary treatment option in pediatric patients with fecalith impaction at the distal colon or rectum, thereby avoiding unnecessary surgical trauma.

## Data Availability

The original contributions presented in the study are included in the article/Supplementary Material, further inquiries can be directed to the corresponding authors.
